# Predictive Factors for Return to Driving After Lower Limb Arthroplasty

**DOI:** 10.1016/j.artd.2025.101685

**Published:** 2025-04-15

**Authors:** Vasileios Giannoudis, Katie Lee, Farag Shuweihdi, Andrew Manktelow, Benjamin Bloch, Bernard van Duren, Hemant Pandit

**Affiliations:** a Leeds Orthopaedic & Trauma Sciences, School of Medicine, University of Leeds, Leeds, UK; b Nottingham Elective Orthopaedic Service, Nottingham University Hospitals NHS Trust, Nottingham, UK; c Medical Statistics & Health Data Science, University of Leeds, Leeds, UK; d Nottingham Elective Orthopaedic Service, Nottingham University Hospitals NHS Trust, Nottingham, UK; e Nottingham Elective Orthopaedic Service, Nottingham University Hospitals NHS Trust, University of Nottingham, School of Medicine, Nottingham, UK; f Leeds Institute of Rheumatic and Musculoskeletal Medicine, University of Leeds, Leeds, UK

**Keywords:** Total hip arthroplasty, Total knee arthroplasty, Driving, Predictive factors

## Abstract

**Background:**

A common question post total hip arthroplasty (THA)/total knee arthroplasty (TKA) arthroplasty is “Doctor, when can I drive?”. No objective assessment currently exists. This study aimed to identify clinical factors predicting driving return post hip THA and TKA.

**Methods:**

In this single-center retrospective observational study, patients were reviewed at 6 weeks post THA and TKA. Patient demographics, driving status, timed up and go (TUG) test, self-reported walking time (SRWT), walking aid use, and pain scores were collected. Descriptive statistics, *t*-tests, and binary regression models were used.

**Results:**

Five hundred ninety two participants were included: 271 THA (males n = 134, mean age: 66.4) and 321 TKA (males n = 155, mean age: 66.8). THA: At 6 weeks, 155 patients (57.1%) were driving and 116 did not drive (DND) (n = 82 female, 70.6%) (*P* < .001). SRWT was longer in driving group (mean 36.35 minutes vs 31.23 minutes [*P* = .072]). TUG tests were faster in driving group (9.51 seconds vs 11.98 seconds [*P* < .001]). Driving inability predictors included using 2 crutches (*P* < .001) and TUG (*P* = .015). TKA: At 6 weeks, 196 patients (61%) were driving and 125 DND (n = 78 female, 62.4%) (*P* < .01). SRWT was longer in driving group (mean 33.6 vs 28.1 minutes [*P* = .31]). TUG tests were faster in driving group (10.18 seconds vs 12.29 seconds [*P* < .001]). Driving inability predictors included “severe” pain scores (*P* ≤ .0001) and >2 walking aids use (*P* = .022).

**Conclusions:**

Following THA/TKA, 60% patients were driving by 6 weeks. Females take longer for driving return. Walking aids negatively impacted driving return, while faster TUG test and longer SRWT were positive predictors.

## Introduction

Total hip arthroplasty (THA) and total knee arthroplasty (TKA) have proven to be 2 of the most effective procedures in improving patient quality of life. In the United Kingdom, approximately 95,000 TKAs and 105,000 THAs are performed annually [1]. The majority of these patients drive a car and therefore a commonly posed question post THA/TKA is “Doctor, when can I return to driving?”.

Multiple studies have shown a significant association between driving cessation and social isolation [2,3]. In turn, social isolation has been associated with several health consequences including earlier mortality and poor mental and physical health [4]. Therefore, being able to provide a clear advice for return to driving is not only important for the patient's safety but also for overall general wellbeing. At present, the advice and level of evidence available for surgeons to provide recommendations are limited. The Driver and Vehicle Licensing Agency in the UK advises that following THA or TKA drivers should follow the process for a “limb disability” requiring completion of documentation which includes physician recommendations and can return to driving when patients feel safe performing an emergency stop [5]. This puts responsibility on the patient who may mean posing danger to themselves or others. Conversely, in the United States, national guidelines were produced by the American Medical Association and National Highway Traffic Safety Administration to quantify safe return to driving post THA/TKA. The following recommendations were made [6]: A person should not drive for at least 4 weeks after right THA. A person should not drive for 3-4 weeks following right TKA.

From a driving safety aspect, the ability to brake in emergencies within sufficient time (Break Reaction Time [BRT]) and applying the necessary force (Break Reaction Force [BRF]) are crucial. Several studies have attempted to quantify the safe return to driving primarily using BRT and less frequently BRF [7,8]. These are frequently assessed using driving simulator models which are costly, cumbersome to install, and not readily available in most orthopaedic centers [[9], [10], [11], [12]]. Other parameters which are easier and cheaper to measure may therefore be more useful. Ideally, a simple clinical test that can be performed by surgeon and/or the patient themselves that can assist with decision-making related to safe return to driving would be of significant benefit to patients and physicians alike.

Historically, both the left and right lower limbs have played a role in driving manual transmission vehicles with the left being used for the clutch pedal and the right for the brake pedal. The use of the left leg in driving has become less important with the increase in automatic transmissions and therefore it has become more important to assess the impact of surgery on the right leg.

This study aimed to identify clinical parameters associated with the ability to return to driving post primary right-sided total hip or total knee arthroplasty.

## Material and methods

At our institute, all lower limb arthroplasty patients are reviewed routinely by a senior specialist lower limb physiotherapist in a joint clinic with the named operating surgeon at 6 weeks postsurgery. At this visit, the following data points are routinely collected: patient demographics, timed up and go (TUG) test, self-reported walking time (SRWT), range of movement (ROM), driving status, current use of walking aids, and self-reported pain scores. In addition, medical or surgical complications which may have impacted return to driving (eg, Debridement, Antibiotics and Implant Retention procedure for suspected infection or patients sustaining postoperative cerebrovascular accidents) were recorded at 6 weeks. This study was a retrospective review of prospectively collected data. Institutional approval for the study was obtained.

Of note, all patients were provided with leaflets preoperatively about their arthroplasty procedures. These leaflets include a specific subsection regarding driving. In both the THA and TKA leaflets, there are the following statements: “You should not drive until you feel able to perform an emergency stop—normally following 6 weeks from your operation. It is your responsibility to check with your insurance company.”

**Figure 1:**
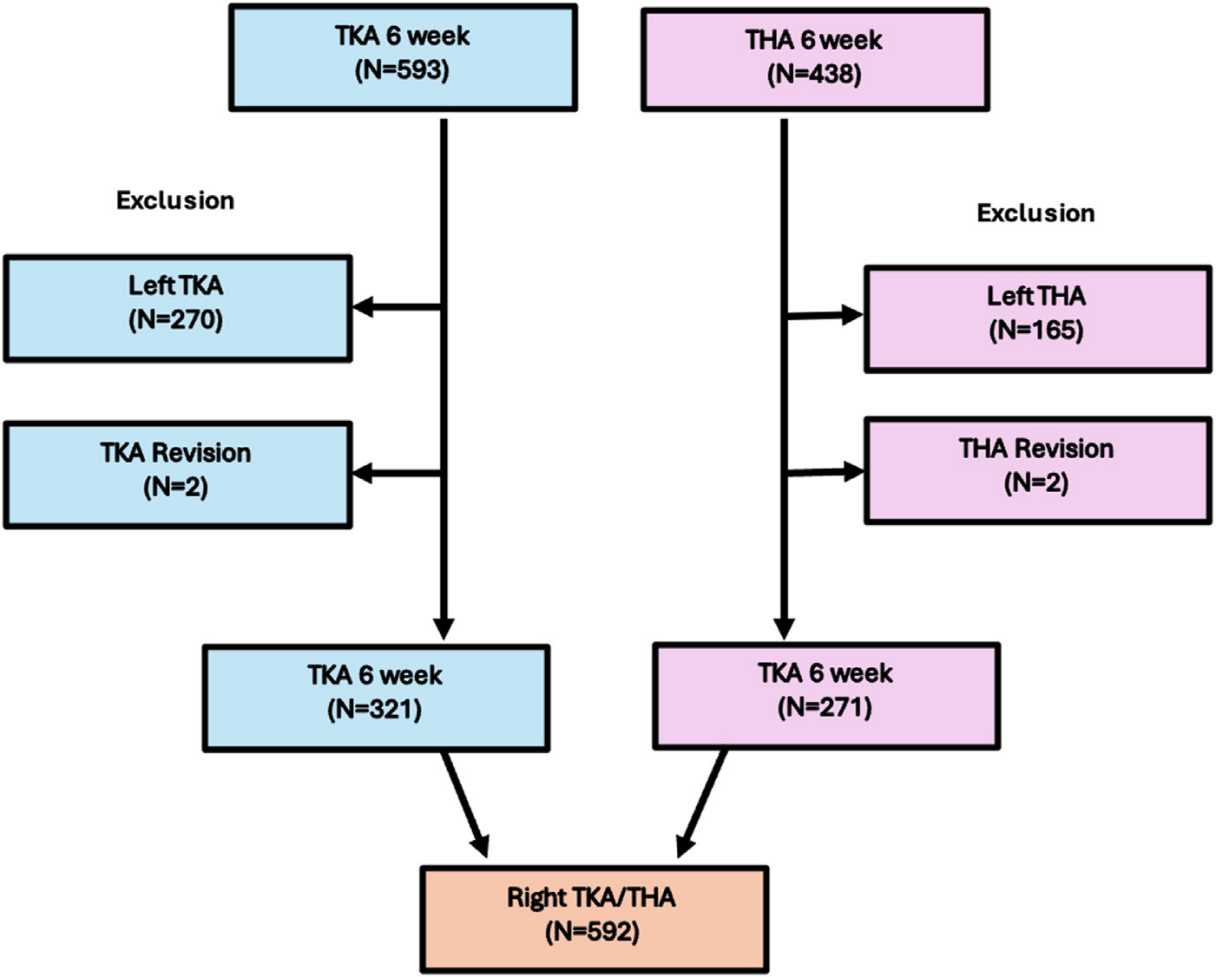
Flow diagram of participant inclusion and exclusion criteria.

For this study, we reviewed all adult patients who reported that they were driving a car prior to their arthroplasty procedure and underwent a primary right-sided THA or TKA between January 2019 and July 2023 and reviewed at 6 weeks postsurgery. Patients undergoing revision hip or knee surgery and non-English speaking individuals were excluded (Fig. 1). Patients were categorized into 2 groups: those who had and those who had not returned to driving (do not drive [DND]).

### Outcome measures

Patient’s age at the time of surgery, sex, type of surgery, and whether the patient held a valid driving license or not were routinely collected. In addition, following assessments were conducted at the 6-week visit.

#### TUG test

The time in seconds was recorded for the patient to stand up from a standard chair with armrests, walk 3 m at a comfortable and safe pace, turn, walk back to the chair, and sit down [13]. Each participant performed the test 3 times, and the average of 3 tests was recorded.

#### SRWT

All patients were asked to report for how long they could walk for before having to stop either due to pain or fatigue. This was documented in minutes. This was based on their longest mobilizing episode during a single day.

#### Range of movement

All ROMs were measured with a goniometer using a standardized technique. For THA, hip flexion and abduction were measured. For TKA, knee flexion and extension were measured.

#### Driving status

All patients were asked at the 6-week mark whether they had returned to driving. This was recorded as “Yes” or “No”.

#### Pain scale assessment

All patients were asked to rank their worst pain from the following options: None, Very mild, Mild, Moderate, and Severe.

#### Use of walking aids indoors/outdoors

All patients were asked to document their use of walking aids indoors and outdoors from the following options: Unaided, 1 stick, 1 crutch, 2 crutches, and frame and wheelchair.

#### Statistical analysis

Statistical analysis was performed using SPSS (Version 29.02.0, APA, Chicago). Continuous data variables were assessed for their data distribution using histograms. Mann-Whitney/Chi-squared test and *t*-test were used for nonparametric/parametric data, respectively. Binary logistic regression modeling was used which used the category “Not driving” and compared it against other parameters. Statistical significance was set at *P* < .05. As part of the post hoc analysis, imputation was used to calculate the missing values for any parameters which were missing more than 2% of data.

## Results

### Demographic information

There were 321 patients who underwent elective right-sided TKA, of which 155 were males (mean age: 68.2; standard deviation [SD]: 9.9) and 166 females (mean age: 66.8; SD: 10.1). At 6 weeks, 196 patients were driving (61%), of which 108 (55%) were males (Table 1).

**Table 1:** Knee analysis summary of knee arthroplasty patient demographics and clinical parameters assessed in 6-week clinical consultation.

	Driving (n = 196): female (n = 88) and male (n = 108)			Not driving (n = 125): female (n = 78) and male (n = 47)			*P* value	Mean rank
	Mean	Median	Standard deviation	Mean	Median	Standard deviation		
Age								
Female (n = 166)	66.3	66	11.3	67.4	67	8.7		
Male (n = 155)	67.0	68.5	8.7	71	72	9.4		
Total (n = 321)	66.7	67.5	10.5	68.8	69	9.1	.097^a^ (_t_1.881 = 290.9)	N/A
TUG (s)								
Female	10.92	9.86	4.17	12.75	11.58	5.40		
Male	9.57	8.81	3.22	11.55	9.80	5.99		
Total	10.18	9.39	3.75	12.29	10.3	5.63	<.001^b^ (Mann-Whitney U 7309.5 s)	
SRWT (min)								
Female	32.1	30	15	26.6	20	15		
Male	34.9	30	16.2	30.5	30	14.7		
Total	33.6	30	16.59	28.10	30	14.9	.314^a^ (_t_-3.11 = 265)	
Flexion (degrees)								
Female	108	110	12.7	104.6	105	12.4		
Male	112.5	110	11.68	109.2	110	10.5		
Total	110.5	110	12.35	106.3	105	11.92	.922^a^ (_t_-3.03 = 271.2)	
Extension (degrees)								
Female	1.21	0	2.6	1.75	0	3.51		
Male	2.59	0	4.24	3.15	0	3.89		
Total	1.97	0	3.67	2.28	0	3.71	.345^a^ (_t_0.725 = 259.9)	

N/A, not applicable; SRWT, self-reported walking time; TUG, timed up and go.

a *t*-test.

b Mann-Whitney U test.

There were 271 patients who underwent elective right-sided THA, of which 134 were males (mean age: 66.4; SD: 11.7) and 137 were females (mean age: 66.1; SD: 11.1). At 6 weeks, 155 patients were driving (57%), of which 100 (64.5%) were males (Table 2).

**Table 2:** Summary of hip arthroplasty patient demographics and clinical parameters assessed in 6-week clinical consultation.

	Driving (n = 155): female (n = 55) and male (n = 100)			Not driving (n = 116): female (n = 82) and male (n = 34)			*P* value
	Mean	Median	Standard deviation	Mean	Median	Standard deviation	
Age							
Female (n = 137)	65.96	66	8.946	66.1	67	12.442	
Male (n = 134)	65.46	66.5	11.789	69.35	69	11.436	
Total (n = 271)	65.64	66	10.838	67.09	67.5	12.194	.0990^a^ (_t_1.532 = 462)
TUG (s)							
Female	9.84	9.00	3.910	11.87	10.40	5.471	
Male	9.34	8.74	3.372	12.22	10.28	6.103	
Total	9.51	8.85	3.567	11.98	10.34	5.650	<.001^a^ (_t_1.881 = 290.9)
SRWT (min)							
Female	37.27	30	17.764	31.87	30	16.369	
Male	35.81	30	16.357	29.71	30	14.717	
Total	36.35	30	16.849	31.23	30	15.861	.018^a^ (_t_-6.32 = 295.5)
Flexion (degrees)							
Female	95	90	7.515	95.06	90	9.226	
Male	93.43	90	11.686	93.82	90	10.155	
Total	93.99	90	10.391	94.69	90	9.484	.24_9_^a^ (_t_-.880 = 428.6)
Abduction (degrees)							
Female	39.91	40	8.136	40.50	40	8.252	
Male	41.26	40	6.599	38.53	40	8.214	
Total	40.78	40	7.189	39.91	40	8.254	.190^a^ (_t_-2.53 = 433.2)

SRWT, self-reported walking time; TUG, timed up and go.

a *t*-test.

At the 6-week review, no patients had encountered any postoperative surgical or medical complications that interfered with their ability to drive. All 592 participants attended their planned 6-week review appointment.

### SRWT

TKA: There was no statistically significant difference in the walking times between the driving (mean: 33.6 minutes; SD: 16.5) and the DND group (mean: 28.1 minutes; SD: 14.9) (*P* = .314). There were trends showing that males were able to walk longer in both the driving (mean: 34.9 minutes; SD: 16.2) and DND group (mean: 30.5 minutes; SD: 14.7 minutes) compared to females (driving: mean 32.1 minutes, SD 15; DND: mean 26.6 minutes, SD 15) (Table 1).

THA: There was a statistically significant difference in the walking times between the driving (mean: 36 minutes; SD: 16.8) and the nondriving group (mean: 31.2 minutes; SD: 15.8) (*P* = .018). Females who were driving (mean: 37.2 minutes; SD: 17.7) could walk slightly longer than their male counterparts (mean: 35.8 minutes; SD: 16.3) and similar trends were also seen in the nondriving group (females [mean: 31.8 minutes; SD: 16.3] vs males [mean: 29.7 minutes; SD: 14.7]) (Table 2).

### TUG

TKA: There was a statistically significant difference in the TUG times between the driving (mean: 10.18 seconds; SD: 3.8) and the nondriving group (mean: 12.29 seconds; SD: 5.6) (*P* ≤ .001). Males were able to complete the TUG test quicker in both the driving (mean: 9.57 seconds; SD: 3.2) and DND group (mean: 11.5 seconds; SD: 6.0 seconds) compared to females (driving: mean 10.9 seconds, SD: 4.2; nondriving: mean 12.75 seconds, SD: 5.4 seconds) (Table 1).

THA: There was a statistically significant difference in TUG times between the driving (mean: 9.51 seconds; SD: 3.6) and the DND group (mean: 11.98 seconds; SD: 5.7) (*P* ≤ .001). Males were able to complete the TUG test quicker in both the driving (mean: 9.34 seconds; SD: 3.4) and DND group (mean: 12.2 seconds; SD: 6.1 seconds) compared to females (driving: mean 9.84 seconds, SD: 3.9; nondriving: mean 11.8 seconds, SD: 5.5 seconds) (Table 2).

### ROM

No significant differences were noted in the ROM at 6 weeks between the driving and DND group for either TKA or THA patients (Table 1 and Table 2).

### Use of walking aids

Post TKA and THA, there was a statistically significant difference between the walking aid requirement indoors and outdoors and return to driving. These results were amplified in the TKA group with 90% of the nondriving cohort reliant on at least 1 crutch at 6 weeks postoperatively when mobilizing outdoors (Table 3). THA patients in the DND group were more likely to be using at least 1 crutch to mobilize (*P* < .0001). These results are summarized (Table 4).

**Table 3:** Summary table showing aids used by patients indoors or outdoors and their driving status post knee arthroplasty.

	Aids	Aids indoors				Aids outdoors			
		Driving	Not driving	Total	*P* value	Driving	Not driving	Total	*P* value
Female	Unaided	82 (94%)	59 (77%)	141 (84%)		47 (53%)	25 (32%)	72 (43%)	
	1 stick	1 (1%)	5 (6%)	6 (4%)		15 (17%)	11 (14%)	26 (16%)	
	1 crutch	3 (3%)	10 (13%)	13 (8%)		17 (19%)	23 (29%)	40 (24%)	
	2 crutches	2 (2%)	3 (3%)	5 (3%)		7 (8%)	16 (20%)	23 (14%)	
	Frame	0	1 (1%)	1 (1%)		2 (3%)	3 (5%)	5 (3%)	
Total		88 (53%)	78 (47%)	166	.029^a^	88 (53%)	78 (47%)	166	.022^a^
Male	Unaided	103 (96%)	37 (79%)	140 (91%)		67 (63%)	15 (33%)	82 (53%)	
	1 stick	2 (2%)	5 (11%)	7 (5%)		17 (16%)	11 (23%)	28 (18%)	
	1 crutch	1 (0.66%)	3 (6%)	4 (2%)		16 (15%)	10 (21%)	26 (17%)	
	2 crutches	1 (0.66%)	2 (4%)	3 (2%)		6 (5%)	11 (23%)	17 (11%)	
	Frame	0	0	0		1 (1%)	0	1 (1%)	
Total		107 (69%)	47 (31%)	154	.007^a^	107 (69%)	47 (31%)	154	.002^a^
Combined	Unaided	185 (95%)	96 (77%)	281 (88%)		114 (58%)	40 (32%)	154 (48%)	
	1 stick	3 (1.5%)	10 (8%)	13 (4%)		32 (16%)	22 (18%)	54 (17%)	
	1 crutch	4 (2%)	13 (10%)	17 (5%)		33 (17%)	33 (26%)	66 (21%)	
	2 crutches	3 (1.5%)	5 (4%)	8 (2%)		13 (7%)	27 (22%)	40 (13%)	
	Frame	0	1 (1%)	1 (1%)		3 (2%)	3 (2%)	6 (1%)	
Total		195 (61%)	125 (39%)	320	<.001^a^	195 (61%)	125 (39%)	320	<.001^a^

a Chi-squared test used.

**Table 4:** Summary table showing aids used by patients indoors or outdoors and their driving status post hip arthroplasty.

	Aids	Aids indoors				Aids outdoors			
		Driving	Not driving	Total	*P* value	Driving	Not driving	Total	*P* value
Female	Unaided	54 (98%)	59 (72%)	113 (83%)		31 (56%)	22 (27%)	53 (39%)	
	1 stick	0	2 (2%)	2 (1%)		7 (13%)	9 (11%)	16 (12%)	
	1 crutch	1 (2%)	12 (15%)	13 (9%)		15 (27%)	31 (38%)	46 (34%)	
	2 crutches	0	3 (4%)	3 (2%)		2 (4%)	14 (17%)	16 (12%)	
	Frame	0	6 (7%)	6 (5%)		0	6 (7%)	6 (4%)	
Total		55 (40%)	82 (60%)	137	.004^a^	55 (40%)	82 (60%)	137	<.001^a^
Male	Unaided	88 (88%)	24 (71%)	112 (84%)		60 (60%)	13 (38%)	73 (54%)	
	1 stick	2 (2%)	4 (12%)	6 (4%)		13 (13%)	3 (9%)	16 (12%)	
	1 crutch	9 (9%)	2 (5%)	11 (8%)		20 (20%)	10 (29%)	30 (22%)	
	2 crutches	1 (1%)	4 (12%)	5 (4%)		6 (6%)	7 (21%)	13 (10%)	
	Frame	0	0	0		1 (1%)	1 (3%)	2 (2%)	
Total		100 (75%)	34 (25%)	134	.050^a^	100 (75%)	34 (25%)	134	<.001^a^
Combined	Unaided	142 (91%)	83 (72%)	225 (83%)		91 (59%)	35 (30%)	126 (46%)	
	1 stick	2 (2%)	6 (5%)	8 (3%)		20 (13%)	12 (10%)	32 (12%)	
	1 crutch	10 (6%)	14 (12%)	24 (9%)		35 (23%)	41 (35%)	76 (28%)	
	2 crutches	1 (1%)	7 (6%)	8 (3%)		8 (4%)	21 (19%)	29 (11%)	
	Frame	0	6 (5%)	6 (2%)		1 (1%)	7 (6%)	8 (3%)	
Total		155 (57%)	116 (43%)	271	<.001^a^	155 (57%)	116 (43%)	271	<.001^a^

a Chi-squared test used.

### Pain scale

TKA: “Pain at best” scores: Overall, more than 70% of the cohort would experience no pain on a settled day. No statistically significant differences were seen in the males (*P* = .903) and female groups (*P* = .601). No statistically significant differences in assessment of “pain at best” scores were seen when reviewing the driving versus DND cohort (*P* = .373).

“Pain at worst” scores: Significant differences were noted with 50% of the DND cohort (n = 125) reporting their pain to be moderate to severe compared to 30% in the driving group (*P* < .001). This was noticed in both females (*P* = .01) and males (*P* = .02) (Table 5).

**Table 5:** Summary table showing self-reported pain scores used by patients. Patients were asked to report pain score at its best and worst at 6 weeks postoperatively post knee arthroplasty.

	Pain at best					Pain at worst			
	Pain score	Driving	Not driving	Total	*P* value	Driving	Not driving	Total	*P* value
Female	None	63 (74%)	55 (71%)	118 (72%)		12 (14%)	2 (3%)	14 (9%)	
	Very mild	6 (7%)	8 (10%)	14 (9%)		6 (3%)	6 (8%)	12 (7%)	
	Mild	15 (17%)	12 (15%)	27 (17%)		38 (45%)	29 (37%)	67 (41%)	
	Moderate	1 (2%)	3 (4%)	4 (2%)		29 (38%)	37 (47%)	66 (41%)	
	Severe	0	0	0		0	4 (5%)	4 (2%)	
Total		85 (52%)	78 (48%)	163	.601^a^	85 (52%)	78 (48%)	163	.011^b^
Male	None	89 (83%)	38 (81%)	127 (82%)		24 (22%)	2 (4%)	26 (17%)	
	Very mild	5 (4%)	3 (6%)	8 (5%)		16 (15%)	2 (4%)	18 (12%)	
	Mild	12 (11%)	5 (11%)	17 (12%)		38 (36%)	23 (49%)	61 (40%)	
	Moderate	1 (1%)	1 (1%)	2 (1%)		27 (25%)	15 (32%)	42 (27%)	
	Severe	0	0	0		2 (2%)	5 (11%)	7 (4%)	
Total		107 (%)	47 (%)	154	.903^a^	107 (69%)	47 (31%)	154	.002^b^
Combined	None	152 (79%)	93 (74%)	245 (77%)		36 (19%)	4 (3%)	40 (13%)	
	Very mild	11 (6%)	11 (9%)	22 (7%)		22 (11%)	8 (6%)	30 (9%)	
	Mild	27 (14%)	17 (14%)	44 (15%)		76 (40%)	52 (42%)	128 (40%)	
	Moderate	2 (1%)	4 (3%)	6 (1%)		56 (29%)	52 (42%)	108 (34%)	
	Severe	0	0	0		2 (1%)	9 (7%)	11 (4%)	
Total		192 (61%)	125 (39%)	317	.373^a^	192 (61%)	125 (39%)	317	<.001^b^

a Fisher's exact test.

b Chi-squared test.

THA: No statistically significant differences were noted between pain at best scores (*P* = .151) or pain at worst scores (*P* = .155). These results are summarized (Table 6).

**Table 6:** Summary table showing self-reported pain scores used by patients. Patients were asked to report pain score at its best and worst at 6 weeks postoperatively post hip arthroplasty.

	Pain at best					Pain at worst			
	Pain score	Driving	Not driving	Total	*P* value	Driving	Not driving	Total	*P* value
Female	None	51 (93%)	74 (90%)	125 (91%)		18 (33%)	29 (35%)	47 (34%)	
	Very mild	1 (2%)	3 (4%)	4 (3%)		14 (25%)	11 (13%)	25 (18%)	
	Mild	3 (5%)	4 (4%)	7 (5%)		15 (26%)	27 (33%)	42 (31%)	
	Moderate	0	1 (2%)	1 (1%)		8 (16%)	14 (17%)	22 (16%)	
	Severe	0	0	0		0	1 (2%)	1 (1%)	
Total		55 (40%)	82 (60%)	137	.580^a^	55 (40%)	82 (60%)	137	.656^a^
Male	None	91 (93%)	31 (91%)	122 (92%)		52 (53%)	19 (56%)	71 (54%)	
	Very mild	2 (2%)	3 (9%)	5 (4%)		15 (15%)	4 (12%)	19 (14%)	
	Mild	5 (5%)	0	5 (4%)		23 (23%)	10 (29%)	33 (25%)	
	Moderate	0	0	0		8 (9%)	0	8 (6%)	
	Severe	0	0	0		0	1 (3%)	1 (1%)	
Total		98 (74%)	34 (26%)	132	.152^a^	98 (74%)	34 (26%)	132	.163^a^
Combined	None	142 (93%)	105 (91%)	247 (92%)		70 (46%)	48 (41%)	118 (44%)	
	Very mild	3 (2%)	6 (4%)	9 (3%)		29 (19%)	15 (13%)	44 (16%)	
	Mild	8 (5%)	4 (3%)	12 (4%)		38 (25%)	37 (32%)	75 (28%)	
	Moderate	0	1 (1%)	1 (1%)		16 (10%)	14 (12%)	30 (11%)	
	Severe	0	0	0		0	2 (2%)	2 (1%)	
Total		153 (57%)	115 (43%)	269	.151^a^	153 (57%)	116 (43%)	269	.155^a^

Patients were asked to report pain score at its best and worst at 6 weeks postoperatively post hip arthroplasty.

a Chi-squared test used.

### Post hoc analysis

Binary logistic regression post imputation of missing datasets (TUG test n = 48; SRWT = 24) was used assessing factors predicting driving return at 6 weeks post TKA or post THA.

TKA: Statistically significant factors were age (odds ratio [OR]: 0.968; *P* = .031), female sex (OR: 1.821; *P* = .031), severe pain (OR: 0.030; *P* < .001), and use of a minimum of 2 crutches outdoors (OR: 0.323; *P* = .022) or 1 stick indoors (OR: 0.049; *P* = .049) (Table 7).

**Table 7:** Binary logistic regression model used to assess impact of various factors on driving return at 6 weeks post knee arthroplasty.

Parameter of interest	Odds ratio	95% CI lower limit	95% CI upper limit	Significance
Age at time of operation	0.968	0.939	0.997	***P* = .031**
Sex (female)	1.821	1.056	3.142	***P* = .031**
Pain at worst (none)	0.416	0.096	1.795	*P* = .236
Pain at worst (mild)	0.253	0.082	0.784	*P* = .018
Paint at worst (moderate)	0.013	0.208	0.061	*P* = .013
Pain at worst (severe)	0.030	0.004	0.235	***P* < .001**
Pain at best (none)	1.205	0.433	3.356	*P* = .721
Pain at best (mild)	2.254	0.932	5.454	*P* = .071
Pain at best (moderate)	1.352	0.114	16.048	*P* = .808
Aids indoors (1 stick)	0.049	0.225	0.051	*P* = .049
Aids indoors (1 crutch)	0.512	0.148	1.767	*P* = .290
Aids indoors (2 crutches)	1.568	0.119	20.696	*P* = .719
Aids outdoors (1 stick)	0.983	0.416	2.324	*P* = .969
Aids outdoors (1 crutch)	0.499	0.247	1.008	*P* = .053
Aids outdoors (2 crutches)	0.323	0.123	0.849	***P* = .022**
Aids outdoors (frame)	1.485	0.152	14.461	*P* = .728
Self-reported walking time	1.005	0.986	1.025	*P* = .603
Knee flexion	1.006	0.983	1.030	*P* = .607
Knee extension	0.982	0.909	1.062	*P* = .650
Timed up & go test	0.964	0.891	1.043	*P* = .355

CI, confidence interval.

Logistic regression model analyzed following data imputation of missing data values.

Bold values indicate significant values (*P* < .05).

THA: Statistically significant factors were female sex (OR: 2.84; *P* < .001), mild pain (OR: 4.3; *P* = .031), use of a minimum of 2 crutches outdoors (OR: 0.248; *P* < .001), and TUG test (OR: 0.91; *P* = .015) (Table 8).

**Table 8:** Binary logistic regression model used to assess impact of various factors on driving return at 6 weeks post hip arthroplasty.

Parameter of interest	Odds ratio	95% CI lower limit	95% CI upper limit	Significance
Age at time of operation	1.001	0.981	1.021	*P* = .941
Sex (female)	2.841	1.881	4.291	***P* < .001**
Pain at worst (none)	1.439	0.789	2.626	*P* = .236
Pain at worst (mild)	0.939	0.575	1.531	*P* = .799
Paint at worst (moderate)	1.227	0.567	2.655	*P* = .603
Pain at worst (severe)	0.010	0.000	-	*P* = 1.000
Pain at best (none)	0.739	0.256	2.132	*P* = .576
Pain at best (mild)	4.366	1.144	16.659	***P* = .031**
Pain at best (moderate)	0.470	0.111	1.988	*P* = 1.000
Aids indoors (1 stick)	1.031	0.463	2.293	*P* = .305
Aids indoors (1 crutch)	1.477	0.243	8.964	*P* = .940
Aids indoors (2 crutches)	0.248	0.011	5.550	*P* = .670
Aids outdoors (1 stick)	0.568	0.287	1.124	*P* = .380
Aids outdoors (1 crutch)	0.321	0.192	0.537	*P* = .104
Aids outdoors (2 crutches)	0.248	0.104	0.593	***P* < .001**
Aids outdoors (frame)	0.624	0.068	5.712	*P* = .677
Self-reported walking time	1.000	0.986	1.015	*P* = .956
Hip flexion	1.002	0.979	1.025	*P* = .893
Hip abduction	1.001	0.970	1.032	*P* = .963
Timed up & go test	0.914	0.850	0.982	***P* = .015**

Logistic regression model analyzed following data imputation of missing data values.

CI, confidence interval.

Logistic regression model analyzed following data imputation of missing data values.

Bold values indicate significant values (*P* < .05).

## Discussion

“Doctor, when can I return to driving?” is a routinely asked question post total hip or knee arthroplasty. This study has identified factors contributing to ability or inability to return to driving post primary right-sided THA/TKA. Presence of pain and use of walking aids at 6 weeks postsurgery predicted inability to return to driving post TKA/THA. TUG test and SRWT are potentially useful adjuncts to aid clinicians in determining a patient's ability to return to driving post THA/TKA. In general, female patients take longer to return to driving than male counterparts.

Self-reported pain scores were used in this study and patients were asked to rate their “pain at its best” and “pain at its worst”. From our study, it appeared that TKA procedures are more painful; however, despite this, there is a slightly higher percentage of patients driving post-TKA (61%) compared to THA (57%). This may be attributed to patients following hip precautions while awaiting a formal pass from their surgeon for return to driving. The use of 2 crutches outdoors is an indicator of an individual's mobility and overall recovery progress following THA/TKA. While the direct prediction of return to driving based on walking aid use has never been directly investigated in the literature, the relationship between mobility recovery and readiness to drive is clear. In our study, the use of 2 crutches outdoors was a strong predictor for inability to drive with patients post THA having a 66% chance of not driving post TKA and 73% chance post THA, respectively. Our study was performed in a temperate climate setting. We feel the use of walking aids was not weather dependent, but due to patient’s instability symptoms postoperatively.

The TUG test is considered a useful assessment to review for patients post TKA/THA [14,15]. For both TKA and THA, the TUG test has excellent inter-rater reliability, and it is quick and easy to perform [16]. Although a clear indicator for THAs, regarding TKA, there are contrasting data published regarding its merits with some studies suggesting it takes months to return to preoperative baseline and others suggesting at 3 weeks patients are faster than their preoperative baseline [14,17]. In our study, we found a significant correlation between quicker TUG test times and driving return. Its use as a surrogate marker for driving return may warrant further investigation.

Our findings show that higher proportions of females take longer to return to driving compared to males. Similar observations have been reported in the literature with higher postoperative pain levels [18], slower functional improvement, and potentially higher complication rates [19] contributing to this delay. While there appears to be limited use of ROM in predicting return to driving, it does have a role in being able to safely get in and out of vehicles. Studies looking at knee flexion post right TKA reviewed minimum knee flexion required to safely enter an estate car (60°) and an SUV car (90°) [20]. This may be useful to measure in clinic to explain to patients the likelihood of them being able to enter and exit their car. There are no studies specifically assessing the range of hip flexion required for entering and exiting vehicles; however, to accelerate or brake, hip flexion needs to be 70° [21]. In our patient cohort, all patients achieved the above parameters (even those who were not driving) and therefore we feel that ROM is unlikely to be a useful parameter in assessing the ability to return to driving post THA/TKA.

Around 60% of the THA/TKA cohort were back to driving by 6 weeks postsurgery. This is in line with the previous studies [9,10]. Most of the published literature uses objective tests like BRT and/or BRF which are difficult to adopt in routine clinical setting and therefore not used.

Due to the retrospective nature of the study, there are several limitations to this study. First, initial pre-hoc power studies to calculate numbers of participants were not performed. However, post-hoc power analysis has shown that the study is adequately powered. We have collected information at a single time point (6 weeks) and therefore not able to demonstrate if in certain patient subgroups it is safe to return to driving before 6 weeks. We have assessed return to driving as self-reported by patients and not whether the patient could drive safely. Furthermore, we do not have the data available of patients' premorbid mobility status which could contribute to the ability to return to driving. However, all these patients had reported their ability to drive preoperatively and it is unlikely that their comorbidities would have changed significantly in a short time. Finally, the lack of preoperative TUG times is also a limitation especially as out study has identified that a faster TUG time is associated with a quicker return to driving.

The study findings are based on real-world data from a large sample of consecutive patients who were assessed using a standardized protocol. This study design has allowed us to identify likely factors which could contribute to a point-of-care testing method to objectively predict the ability to return to driving, an important functional requirement to re-establish patients' independence. The rationale for use of the right limb alone in this study is its key role in braking and the global shift being from manual to automatic transmission use. In America, 98% of vehicles use an automatic transmission and in recent decades there has been a significant surge in automatic transmission vehicles in Europe [22]. This will be accelerated further with global aims to increase electric vehicles use which are also run on automatic transmission motors.

## Conclusions

In summary, this study has identified parameters correlating with return to driving and these potentially can be used as surrogate markers for predicting driving return. Ultimately, we propose the development of a validated patient-individualized scoring system to predict the likelihood of return to driving post right THA/TKA. This should be easy to perform and accessible for clinicians and patients. If there are concerns regarding driving status, we would recommend referral of patients for formal driving assessments. The presence of a simple and validated clinician assessment would provide clarity for all key stake holders involved.

## Funding

The authors are supported in part by the National Institute for Health and Care Research (NIHR) Leeds Biomedical Research Centre (BRC) (NIHR203331). The views expressed are those of the authors and not necessarily those of the National Health Service (NHS), the NIHR, or the Department of Health and Social Care.

## Conflicts of interest

Andrew Manktelow received royalties from Matortho; received speakers bureau/paid presentations for Zimmer Biomet and Medacta; is a paid consultant for Matortho, Zimmer Biomet, and Medacta; received research support from Matortho as a Principal Investigator; and is the Council member of the British Orthopaedic Association. Benjamin Bloch received speakers bureau/paid presentations for Johnson & Johnson and Zimmer Biomet; is a paid consultant for Johnson & Johnson and Zimmer Biomet; and is in the Bone & Joint 360 Editorial Board. Bernard van Duren received research support from Medacta International and received NIHR BRC funding as a Principal Investigator and is the editorial board member of BJJ 360. Hemant Pandit is a paid consultant for Medacta International, Zimmer Biomet, Allay Therapeutics, Paradigm Pharmaceuticals, MATOrtho, Microport, Invibio, Teleflex, and Grunenthal; holds stock or stock options in Allay Therapeutics; received research support from Medacta International, Zimmer Biomet, Allay Therapeutics, and Paradigm Pharmaceuticals as a Principal Investigator; received institutional support for research from Allay Therapeutics, Zimmer Biomet, Paradigm Pharmaceuticals, Invibio, and Depuy Synthes; and is the member of Clinical Advisory Board for Allay Therapeutics. All other authors declare no potential conflicts of interest.

For full disclosure statements refer to https://doi.org/10.1016/j.artd.2025.101685.

## CRediT authorship contribution statement

**Vasileios Giannoudis:** Writing – original draft, Formal analysis, Data curation. **Katie Lee:** Writing – review & editing, Data curation. **Farag Shuweihdi:** Methodology. **Andrew Manktelow:** Writing – review & editing, Supervision. **Benjamin Bloch:** Writing – review & editing, Supervision, Conceptualization. **Bernard van Duren:** Writing – review & editing, Supervision. **Hemant Pandit:** Writing – review & editing, Supervision.

## Appendix A Supplementary data

Conflict of Interest Statement for Manktelow

Conflict of Interest Statement for van Duren

Conflict of Interest Statement for Bloch

Conflict of Interest Statement for Shuweihdi

Conflict of Interest Statement for Pandit

Conflict of Interest Statement for Lee

Conflict of Interest Statement for Giannoudis
