# Supplementary material for: Predictive Factors for Return to Driving After Lower Limb Arthroplasty
Source: Arthroplast Today. 2025 Apr 15;33:101685. doi: 10.1016/j.artd.2025.101685 (PMC12044195; doi:10.1016/j.artd.2025.101685)
Supplement: Conflict of Interest Statement for Bloch [file mmc3.docx]

CONFLICT OF INTEREST STATEMENT Arthroplasty Today

(Adopted from the American Academy of Orthopaedic Surgeons disclosure statement)

The following form **must be ﬁlled out completely and submitted by each author (example, 6 authors, 6 forms). If no discloser is required, please write/type “none” at the end of each sentence.**

Manuscript Title: Predictive factors for return to driving after lower limb arthroplasty.

1. Royalties from a company or supplier (The following conﬂicts were disclosed)

NONE

2. Speakers bureau/paid presentations for a company or supplier (The following conﬂicts were disclosed)

Johnson & Johnson, ZimmerBiomet

3A. Paid employee for a company or supplier (The following conﬂicts were disclosed)

None

3B. Paid consultant for a company or supplier (The following conﬂicts were disclosed)

Johnson & Johnson, ZimmerBiomet

3C. Unpaid consultants for a company or supplier (The following conﬂicts were disclosed)

None

4. Stockor stockoptionsina company or supplier (Thefollowing conﬂictswere disclosed)

None

5. Research support from a company or supplier as a Principal Investigator (The following conﬂicts were disclosed)

None

6. Other ﬁnancial or material support from a company or supplier (The following conﬂicts were disclosed)

None

7. Royalties, ﬁnancial or material support from publishers (The following conﬂicts were disclosed)

None

8. Medical/Orthopaedic publications editorial/governing board (The following conﬂicts were disclosed)

Bone & Joint 360 Editorial Board

9. Board member/committee appointments for a society (The following conﬂicts were disclosed)

None

EachauthormustsignANDprintortypehis/hername,dateandsubmitaseparateform.

In addition, one BLINDED Conﬂict of Interest form (no author names used) should be submitted per manuscript with all author disclosures.


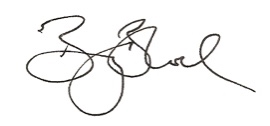


Benjamin Bloch 14/11/2024

Author Name (Print or Type) Author Signature Date
