# Supplementary material for: Predictive Factors for Return to Driving After Lower Limb Arthroplasty
Source: Arthroplast Today. 2025 Apr 15;33:101685. doi: 10.1016/j.artd.2025.101685 (PMC12044195; doi:10.1016/j.artd.2025.101685)
Supplement: Conflict of Interest Statement for Pandit [file mmc5.docx]

CONFLICT OF INTEREST STATEMENT Arthroplasty Today

(Adopted from the American Academy of Orthopaedic Surgeons disclosure statement)

The following form **must be ﬁlled out completely and submitted by each author (example, 6 authors, 6 forms). If no discloser is required, please write/type “none” at the end of each sentence.**

Manuscript Title: Predictive factors for return to driving after lower limb arthroplasty.

1. Royalties from a company or supplier (The following conﬂicts were disclosed)

None

2. Speakers bureau/paid presentations for a company or supplier (The following conﬂicts were disclosed)

None

3A. Paid employee for a company or supplier (The following conﬂicts were disclosed)

None

3B. Paid consultant for a company or supplier (The following conﬂicts were disclosed)

Medacta International, Zimmer Biomet, Allay Therapeutics, Paradigm Pharmaceuticals, MATOrtho, Microport, Invibio, Teleflex and Grunenthal

3C. Unpaid consultants for a company or supplier (The following conﬂicts were disclosed)

None

4. Stockor stockoptionsina company or supplier (Thefollowing conﬂictswere disclosed)

Allay Therapeutics

5. Research support from a company or supplier as a Principal Investigator (The following conﬂicts were disclosed)

Medacta International, Zimmer Biomet, Allay Therapeutics, Paradigm Pharmaceuticals

6. Other ﬁnancial or material support from a company or supplier (The following conﬂicts were disclosed)

Institutional support for research from Allay Therapeutics, Zimmer Biomet, Paradigm Pharmaceuticals, Invibio, Depuy Synthes

7. Royalties, ﬁnancial or material support from publishers (The following conﬂicts were disclosed)

None

8. Medical/Orthopaedic publications editorial/governing board (The following conﬂicts were disclosed)

None

9. Board member/committee appointments for a society (The following conﬂicts were disclosed)

Member of Clinical Advisory Board for Allay Therapeutics

EachauthormustsignANDprintortypehis/hername,dateandsubmitaseparateform.

In addition, one BLINDED Conﬂict of Interest form (no author names used) should be submitted per manuscript with all author disclosures.


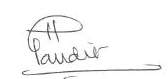


PROF HEMANT PANDIT 14/11/2024

Author Name (Print or Type) Author Signature Date
